# Comparisons of magnetic resonance imaging, histopathological and Ki-67 labeling index findings in a single myxofibrosarcoma: a case report

**DOI:** 10.1186/s13256-024-04693-y

**Published:** 2024-08-16

**Authors:** Ryusuke Tsujimura, Kenta Uto, Noriyuki Nakano, Yusuke Sato, Yuko Kobashi, Toshio Kojima, Hiroyuki Hao

**Affiliations:** 1 Division of Human Pathology, Department of Pathology and Microbiology, Nihon University School of Medicine, 30-1, Oyaguchi Kami-Chou, Itabashi-Ku, Tokyo, 173-8610 Japan; 2 Department of Orthopaedic Surgery, Nihon University School of Medicine, 30-1, Oyaguchi Kami-Chou, Itabashi-Ku, Tokyo, 173-8610 Japan; 3 Department of Radiology, Nihon University School of Medicine, 30-1, Oyaguchi Kami-Chou, Itabashi-Ku, Tokyo, 173-8610 Japan

**Keywords:** Myxofibrosarcoma, Ki-67, Tail sign, Cellular portions, Myxoid portions

## Abstract

**Background:**

Myxofibrosarcoma is a myxoid soft tissue sarcoma showing T2 high intensity on magnetic resonance imaging. However, myxofibrosarcoma is a heterogeneous sarcoma with both myxoid and cellular portions. Magnetic resonance imaging findings were obtained MRI findings for comparison with histological and Ki-67 immunohistochemical features, in different portions of one myxofibrosarcoma.

**Case presentation:**

Magnetic resonance imaging observations were compared with gross pathological and microscopic findings of a myxofibrosarcoma from a 50-year-old Japanese female. The Ki-67 labeling indices of different portions of the tumor, that is, the myxoid, cellular, and histologically confirmed infiltrative margin portions (pathological tail sign), were compared. The T2 low intensity area was more cellular than the T2 high intensity area, while the cellular portion had a significantly higher Ki-67 index than the myxoid portion (*p* = 0.0313). The portions with the pathological tail sign had a significantly higher Ki-67 labeling index than those without this sign (*p* = 0.0313).

**Conclusions:**

More cellular portions of a myxofibrosarcoma correspond to more areas of the tumor showing aggressive features. Furthermore, our data also support the hypothesis of high aggressiveness being associated with the pathological tail sign in myxofibrosarcoma. To our knowledge, this is the first case report to describe comparisons among the imaging findings, histological features, and Ki-67 immunohistochemistry results for different portions of one myxofibrosarcoma.

## Background

Myxofibrosarcoma is a myxoid soft tissue sarcoma showing T2 high intensity on magnetic resonance imaging (MRI). However, it is noteworthy that myxofibrosarcoma is a heterogeneous sarcoma with both myxoid and cellular portions. We compared the imaging, histological, and immunohistochemical findings of a single myxofibrosarcoma. Ki-67 protein is known to be highly expressed, immunohistochemically, in cycling cells but was strongly downregulated in resting G0 cells. This characteristic has made Ki-67 a clinically important proliferation marker for the grading of various cancers [1]. We herein describe our experience with a myxofibrosarcoma patient. To our knowledge, this is first case report to compare MRI findings, histological features, and Ki-67 immunohistochemistry results for different portions of one myxofibrosarcoma.

## Case presentation

The patient was a 50-year-old Japanese female. She had initially noticed a mass in her right lower limb. One year after her initial visit to a hospital for evaluation of the mass, she was referred to Nihon University Itabashi Hospital. Clinically, the mass was 6.5 cm × 5.5 cm × 2.5 cm in size and movable, elastic and hard, with no tenderness. Skin erosion was observed at the center of the mass. A biopsy conducted at our hospital yielded a pathological diagnosis of myxofibrosarcoma. Experienced surgeons performed wide resection of the tumor at Nihon University Itabashi Hospital. The margin of resection was clear except for the deep margin.

**Fig. 1:**
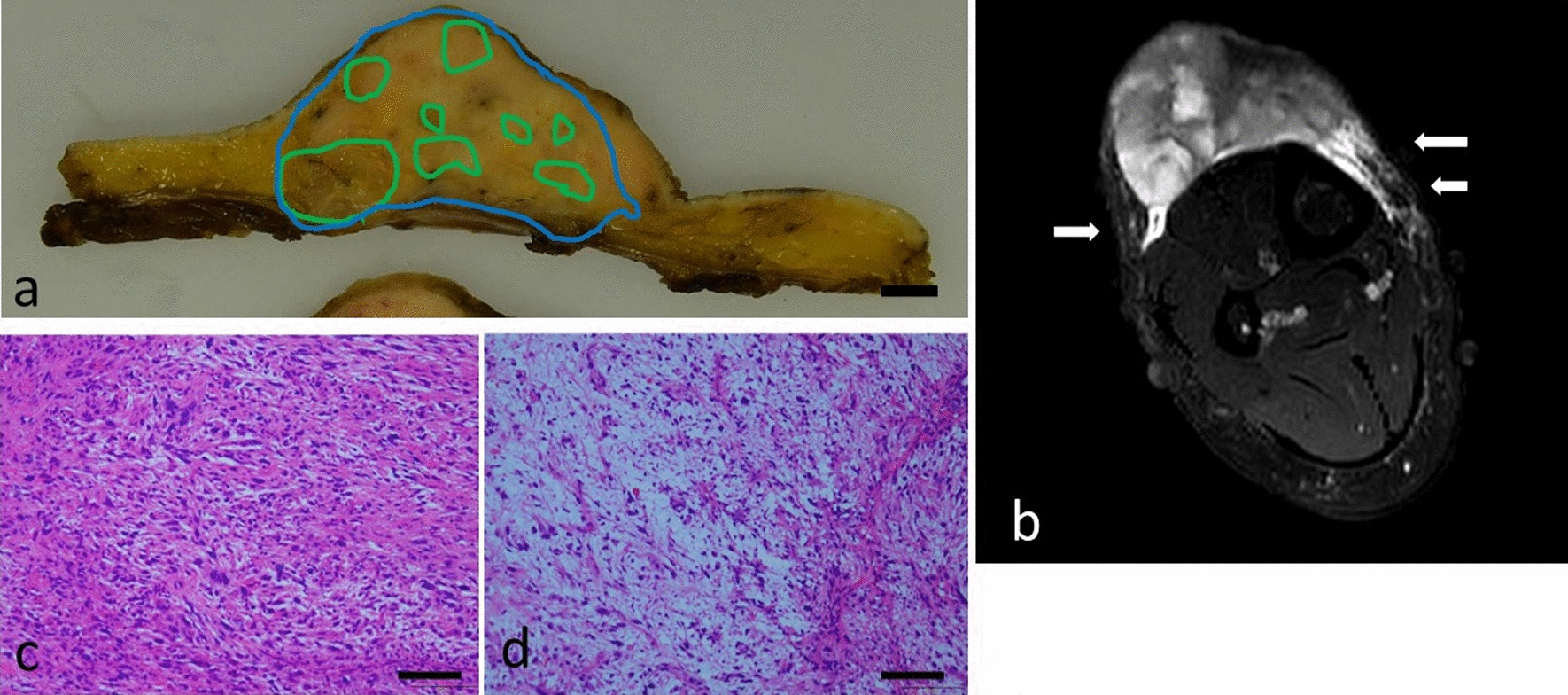
**a** Horizontal gross section of the surgically resected specimen. The tumor area is surrounded by a blue line. The myxoid areas are surrounded by a green line. Other areas of the tumor, not shown with green lines, are the cellular portions. These areas were confirmed histologically (bar is 1 cm). **b** Horizontal section of T2-weighted magnetic resonance imaging (MRI) with fat suppression of the same section as in **a**. Tail signs are seen (arrow). **c** Cellular portion of the tumor, not surrounded by a green line as in **a** (×100; bar, 200 μm). **d** Myxoid portion of the tumor, surrounded by a green line as in **a** (×100; bar, 200 μm)

An MRI of her leg revealed areas with T1 iso, T2 low, and T2 high intensities in the tumor with an infiltrative myxoid margin (tail sign) on the left and right sides of a horizontal section of the tumor (Fig. 1b).

Grossly, the totally resected specimen was a 6 cm × 5 cm × 2.8 cm multinodular tumor beneath the skin. Distinguishing between the myxoid and cellular portions was difficult, and the tumor margin was smooth rather than infiltrative (Fig. 1a). Microscopically, pleomorphic to spindle cells had proliferated, creating a lobulated appearance with myxoid stroma, alternating hypocellular myxoid areas and hypercellular areas (Fig. 1c,d). The pathological diagnosis was myxofibrosarcoma.

Comparing the imaging and pathological findings revealed that the T2 low intensity area corresponded to the cellular portion on histological sections (Fig. 1c), while the T2 high intensity area corresponded to the myxoid portion (Fig. 1d).

The cellular portions of the tumor showing T2 low intensity on MRI were larger than the myxoid portions. The tail sign was observed on MRI, but on the histological sections, the left side of the tumor showed a protrusion corresponding to a tumorous component only 5 mm in size (pathological tail sign). On the right side, the tumor had a smooth contour histologically, and there was no infiltrative margin. Bilaterally, the main area showing the tail sign on MRI was subcutaneous tissue without tumor components on histological sections. The pathological tail sign was subtle as compared with that observed on MRI.

For all statistical analyses, EZR32 (Saitama Medical Center, Jichi Medical University, Saitama, Japan), which is a graphical user interface for R (The R Foundation for Statistical Computing, Vienna, Austria), was used to compare Ki-67 labeling index values among different portions of the tumor. The Wilcoxon signed-rank exact test was applied for analysis of the data. For the Ki-67 labeling index cell counts, we used “e-count” software (Biomedical Science Co., Ltd., Tokyo, Japan).

The Ki-67 labeling index was determined in myxoid, cellular, and pathological tail sign portions of the tumor, at five sites in each portion, with 400× magnification, microscopically. The Ki-67 labeling index values were determined (cellular areas: 30.25 ± 3.47%, myxoid parts: 15.77 ± 3.78%, pathological tail sign parts: 38.45 ± 11.96%).

**Fig. 2:**
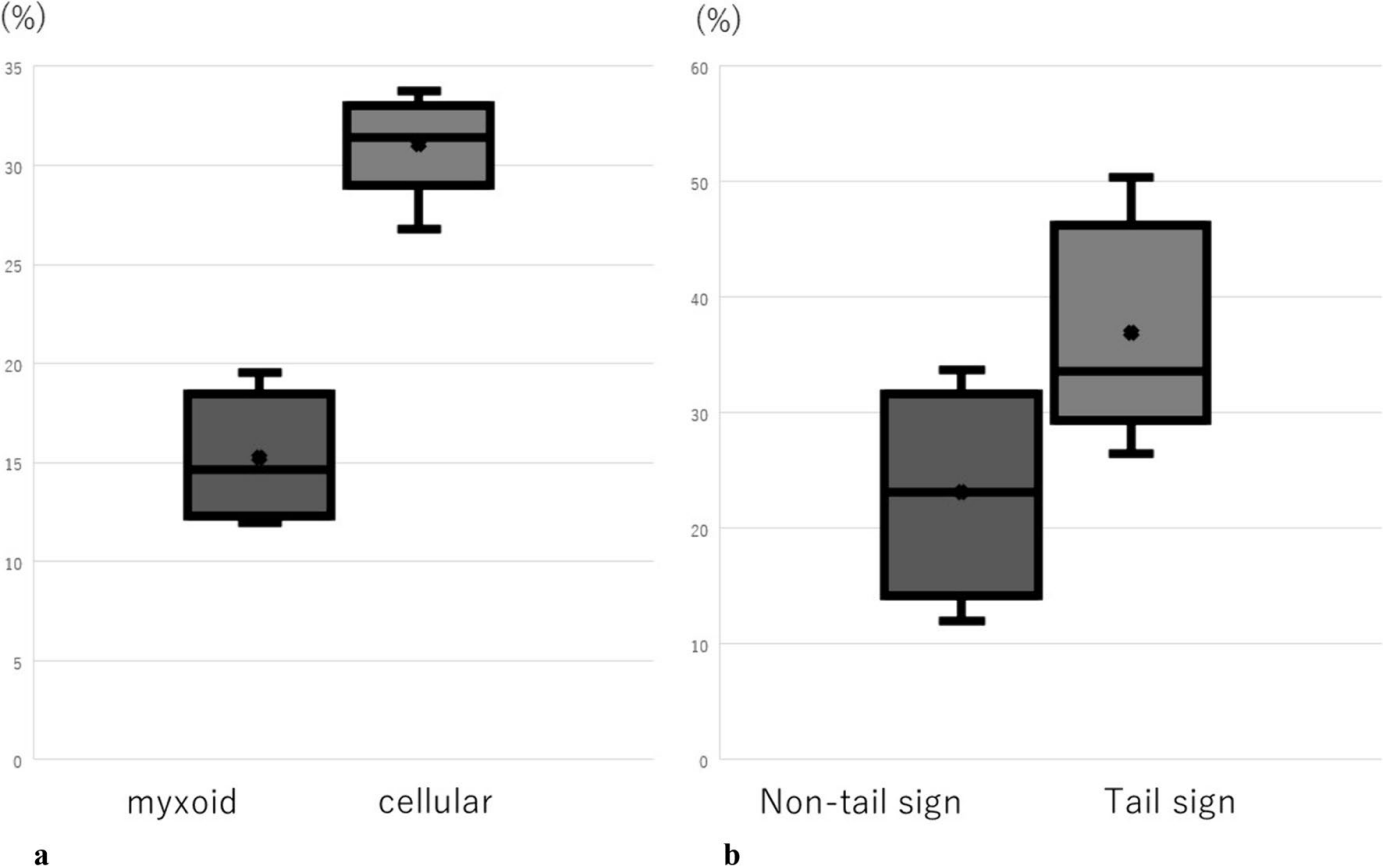
**a** Comparison of Ki-67 labeling indexes in the myxoid and cellular portions of the tumor. The Ki-67 labeling index is significantly higher in the cellular than in the myxoid portions of the tumor (*p* = 0.0313). **b** Comparisons of Ki-67 labeling indices in portions of the tumor with and without the pathological tail sign. The Ki-67 labeling index is significantly higher in the portions of the tumor with than in those without the pathological tail sign (*p* = 0.0313)

The Ki-67 labeling index difference between the five cellular sites and the five myxoid sites was statistically significant (*p* = 0.0313), with the Ki-67 labeling index being higher in cellular than in the myxoid portions of the tumor (Fig. 2a). On MRI, the cellular portion was T2 low and the myxoid portion was T2 high. Consequently, the T2 low portion had a high Ki-67 labeling index, while the Ki-67 labeling index was low in the T2 high portion. Examination of Ki-67 labeling indices at five sites in portions of the tumor with the pathological tail sign and ten sites without this sign (both cellular and myxoid) showed these indices to be significantly higher in portions of the tumor with than in those without the pathological tail sign (*p* = 0.0313) (Fig. 2b).

The patient received radiation therapy postoperatively, due to proximity of the deep side of the margin. The patient remains alive without recurrence 11 months after the operation.

## Discussion

Giulia *et al*. [2] reported that, based on radiological findings, a very high content of myxoid matrix accounts for the “water-like” appearance of myxofibrosarcoma, which shows a very high signal on fluid-sensitive sequences, and, furthermore, when the “water-like” appearance is more intense, there is a greater risk of local recurrence after excision [2]. In our present case, according to the Ki-67 labeling index, the cellular portions of the tumor had significantly more aggressive features than the myxoid portions. This observation does not support the aforementioned hypothesis of Giulia and colleagues. However, pathologically, cellular myxofibrosarcoma is generally considered to be a more high-grade tumor than myxoid myxofibrosarcoma [3, 4]. Though controversial, there appears to be a difference in the rate of local recurrence between the cellular and myxoid forms of myxofibrosarcoma [4]. Our findings in the current case suggest that the more cellular a myxofibrosarcoma is, the more aggressive tumor portions it contains.

The tail sign is based mainly by imaging appearance, having been pathologically defined in only a few reports, one of which described it as tapered fascial enhancement extending from the tumor margin exceeding 2 mm in thickness [5]. In our present patient, the infiltrative margin was observed as a 5 mm tumor protrusion on histological sections (pathological tail sign). It was subtle as compared with the tail sign observed on MRI. Using MRI, Spinnato *et al*. [6] found the tail sign to be the second most valuable independent predictor, after tumor size, of local recurrence after excision. However, in our case, most of the area showing the tail sign on MRI was subcutaneous tissue with no histological evidence of tumor components. When determining surgical margins to avoid excessive tissue removal, decisions should be based on combining MRI with other approaches such as positron emission tomography. The Ki-67 labeling index in our present case was significantly higher in the portions of the myxofibrosarcoma showing the pathological tail sign than in those without this sign. This observation supports the pathological tail sign in myxofibrosarcoma being a highly aggressive feature of this tumor.

## Conclusion

This case report is the first, to our knowledge, to examine the tumor aggressiveness of different portions of a single myxofibrosarcoma based on the Ki-67 labeling index. Our findings indicate that the more cellular a myxofibrosarcoma is, the more aggressive tumor components it tends to contain. Furthermore, the pathological tail sign in myxofibrosarcoma appears, as previously suggested, to indicate a highly aggressive tumor.

## Data Availability

All data generated or analyzed during preparation of this case report are included in this published article and the associated supplementary information.
